# Prevalence and etiologies of pulmonary hypertension at Somalia-Turkey Training and Research Hospital in Mogadishu

**DOI:** 10.11604/pamj.2021.40.215.30159

**Published:** 2021-12-09

**Authors:** Gökhan Alıcı, Ömer Genç

**Affiliations:** 1 Department of Cardiology, Turkey Recep Tayyip Erdogan, Somalia Mogadishu Training and Research Hospital, Mogadishu, Somalia,; 2 Department of Cardiology, Ağri Training and Research Hospital, Ağri, Turkey

**Keywords:** Pulmonary hypertension, epidemiology, Somalia

## Abstract

**Introduction:**

pulmonary hypertension (PH) is one of the leading causes of mortality in the world. This study aimed to determine the ratio distribution and etiological characteristics of PH in Somalia-Turkey Training and Research Hospital.

**Methods:**

the study was designed as a hospital-based retrospective observational study and included 260 patients who were diagnosed with PH by transthoracic echocardiography (TTE) in the cardiology outpatient clinic in Somalia-Turkey Training and Research Hospital in Mogadishu. Sociodemographic and clinical characteristics and data on morbidity were retrieved from clinical records.

**Results:**

the echocardiographic prevalence of PH was found to be 18% (n=260). PH due to left heart disease was the most common form of PH (n=151, 58%), followed by PH due to lung disease (n=61, 23%), group 1 PH (n=38, 16%), group 5 PH (n=7, 2%), and chronic thromboembolic PH (CTEPH) (n=3, 1%).

**Conclusion:**

the present study showed that the prevalence of PH in Somalia is relatively higher than the rest of the world's average. The distribution characteristics of the disease could be related to the region-specific differences of the causative diseases. Further studies are needed to better capture the epidemiology of PH in Somalia.

## Introduction

Pulmonary hypertension (PH) is a heterogeneous group of diseases that leads to impaired health-related quality of life and early mortality, if untreated. In case of clinical suspicion, transthoracic echocardiography (TTE) is helpful in the diagnosis, while right heart catheterization is the gold standard diagnostic method. In 6^th^ world symposium on pulmonary hypertension (Nice, France, 2018), a mean pulmonary artery pressure (PAP) of >20mmHg in healthy individuals was recommended as the limit for the definition of PH [1]. In PH, which is a significant cause of morbidity and mortality, effective and robust solutions can be attained by adopting the same terminology with a multidisciplinary approach. The most recent update in the clinical classification of pulmonary hypertension was made in 6^th^ world symposium on PH. The basic principle of clinical classification is to categorize diseases with similar physiopathological mechanisms, clinical findings, hemodynamic features, and treatments under a common topic. PH is clinically classified under five groups: i) pulmonary arterial hypertension; ii) PH due to left heart disease; iii) PHdue to chronic lung disease and/or hypoxia; iv) chronic thromboembolic PH (CTEPH) and v) PH due to unclear multifactorial mechanisms [2].

The prognosis of PH is related to these clinical groups, namely, the survival of PH due to left heart disease and CTEPH are better than that of pulmonary arterial hypertension (PAH) cases (group 1), and the worst prognosis is in PH due to lung diseases, such as chronic obstructive pulmonary disease (COPD) and idiopathic pulmonary fibrosis (IPF) [3]. Because of the variability of etiological factors, the prevalence of PH in African countries is higher than in developed countries [4,5]. Studies on mortality in a small number of countries on the continent have shown that PH is the leading cause of death [6,7]. Somalia, where our study was conducted, has a special place among African countries; in this country, there is no social security system and social state policy due to the ongoing civil war [8]. The healthcare system is completely run by private healthcare institutions and all the transactions are performed without supervision. Access to medicine, treatment, and necessary tests is unequal and difficult for chronic patients. Accordingly, data on PH in Somalia is limited and, to our knowledge, there has been no comprehensive study on the distribution of PH in the country. The aim of this study was to determine the distribution and etiological characteristics of PH at the Somalia-Turkey Training and Research Hospital in Mogadishu Somalia.

## Methods

**study design and setting:** the study was designed as a hospital-based retrospective observational study and included 260 patients who were diagnosed with PH by transthoracic echocardiography (TTE) in the cardiology outpatient clinic in Somalia-Turkey Training and Research Hospital in Mogadishu, the capital city of Somalia. This institution is the largest and only referral hospital in the country where graduate-level physician assistant programs are administered and advanced cardiac tests and treatments are performed. Sociodemographic and clinical characteristics including age, gender, body mass index (BMI), chronic drug use, human immunodeficiency virus (HIV) status, history of systemic hypertension, history of sickle cell anemia, history of tuberculosis, history of dyslipidemia, history of other morbidities, cigarette consumption, and thoracic computerized tomography (CT) images were retrieved from clinical records or via telephone interviews made with patients who had incomplete medical records. Ethical approval was obtained from the only local ethics committee in the region. The need for informed consent was waived due to retrospective nature of the study.

**Echocardiographic assessment:** echocardiographic evaluation was performed using a Toshiba Aplio 500 device with a 3-5Mhz probe. Echocardiograms were obtained from the standard parasternal, apical, and subcostal windows. All the evaluations were performed by M-mode, 2-dimensional (2D), and Doppler echocardiography. Echocardiograms were examined and approved by a cardiologist specialized in Turkey. In echocardiographic examination, PH was defined as right ventricular systolic pressure (RVSP) higher than 35mmHg without acute right heart failure and pulmonary stenosis. Left ventricular ejection fraction (LVEF), systolic pulmonary artery pressure (sPAP), interventricular septum wall thickness, diastolic functions, and degenerative and rheumatic mitral valve disease were noted in echocardiographic examination. All the echocardiographic examinations were performed according to American Society of Echocardiography (ASE) guidelines [9]. Pulmonary artery systolic pressure was measured using the bernoulli equation with tricuspid regurgitant velocity (TRV) and right atrial pressures. Right atrial pressures were calculated using the size of the inferior vena cava (IVC) and the degree of collapsibility. Significant valvular disease was defined as moderate-to-severe stenosis or regurgitation. Left ventricular EF (LVEF) was calculated using the Simpson method. Tricuspid annular plane systolic excursion (TAPSE) was used to detect right ventricular systolic dysfunction. Rheumatic valvular disease was detected by valvular morphology on 2D echocardiography and Doppler ultrasound evaluations. Pericardial effusion greater than 5mm was considered significant. In addition, presence of congenital heart disease, vegetation, pace leads, and thrombi were recorded during echocardiographic evaluation.

**Statistical analysis:** data were analyzed using IBM SPSS for Windows version 20.0 (Armonk, NY: IBM Corp.). Normal distribution of continuous variables was assessed using analytical (Kolmogorov-Smirnov test) and visual methods (histograms and probability plots). Continuous variables were expressed as mean ± standard deviation (SD) or median interquartile range (IQR) and categorical variables were expressed as frequency (n) and percentage (%). Continuous variables were compared using the Mann-Whitney U test and student T test, as appropriate. Categorical variables were compared using Chi-square and Fisher´s exact tests. A two-tailed p-value of <0.05 was considered significant throughout the analysis.

**Ethical considerations:** the study was approved by the ethics committee of Mogadishu Somalia-Turkey Recep Tayyip Erdogan Training and Research Hospital (date: 24.02.2021, decision no: 328, reference no: MSTH/5289).

## Results

A total of 1428 patients underwent echocardiographic examination in our clinic within the six-month period between July and December 2020. Of these, 260 patients with TRV greater than 3m/sec and/or sPAP higher than 35mmHg were included in the study. Overall, the prevalence of PH was 18.2% (n=260). In these patients, the prevalence of PH was 18.2% (n=260).

**Figure 1:**
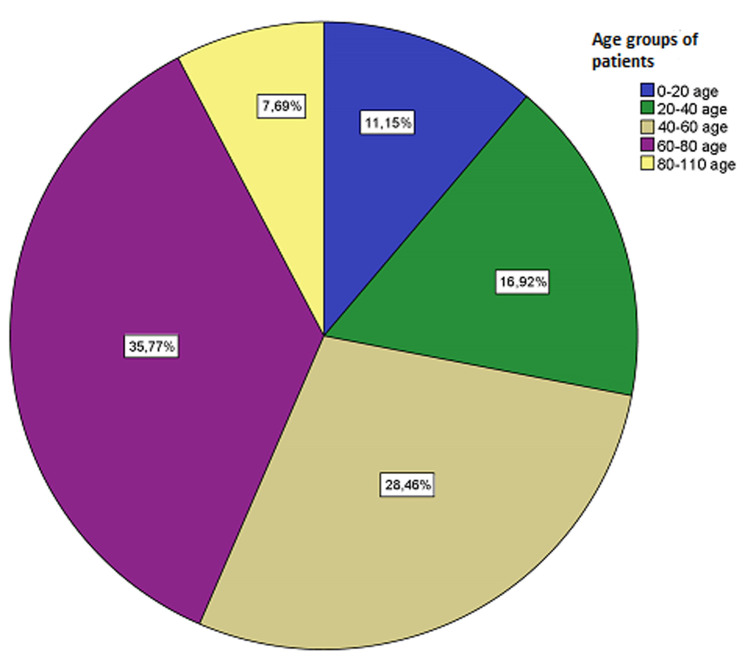
case distribution by age groups

**Demographic and clinical characteristics:** mean age of the participants was 53±17.51 years and most of them were male 57.3% (n=149). All the patients 100% (n=260) were black Africans. **Figure 1** presents the distribution of cases by age groups. Most common age group was 60-80 (35.7%) years, followed by 40-60 (28.4%) years. Most frequent comorbidity was heart failure (44.6%). Of all patients, 5% of the patients were active smokers and three patients were present with HIV. Table 1 presents baseline characteristics of the patients.

**Table 1:** baseline demographic and clinical characteristics of patients

Variable	All patients(N=260)
**Demographics**	
Age (years)	53±17.51
**Gender n (%)**	
Females	111(42.7)
Males	149(57.3)
**Race**	
Blacks n (%)	260 (100)
**Comorbidities**	
Systemic arterial hypertension n (%)	55(21.2)
Diabetes mellitus n (%)	60 (23.1)
Smoking n (%)	13(5.0)
Heart failure n (%)	116(44.6)
Tuberculosis n (%)	46(17.9)
Chronic obstructive pulmonary disease n (%)	15(5.8)
Human immunodeficiency virus n (%)	3(1.2)

**Echocardiographic findings:** median pulmonary artery systolic pressure (PASP) was 56mmHg (IQR: 50-65mmHg), and mean TAPSE was 16mm (IQR: 14-19mm). The EF was below 45% in 44.6%, significant pericardial effusion was detected in 38.4%, and congenital heart disease was present in 13.1% of the patients (Table 2).

**Table 2:** echocardiographic findings of patients

Variable	All patients (N=260)
Tricuspid regurgitant velocity(m/sec), median(IQR)	3.22(3.12-3.56)
Pulmonary artery systolic pressure (mmhg), median(IQR)	56(50-65)
Tricuspid annular plane systolic excursion(mm), median(IQR)	16(14-19)
Left ventricular ejection fraction (%), median(IQR)	55(3-60)
Left ventricular ejection fraction<45%	116(44.6)
Congenital heart disease, n (%)	34(13.1)
Pace lead, n (%)	3(1.2)
Left ventricle thrombi, n (%)	5(1.9)
Pericardial effusion n (%)	100(38.4)

**Figure 2:**
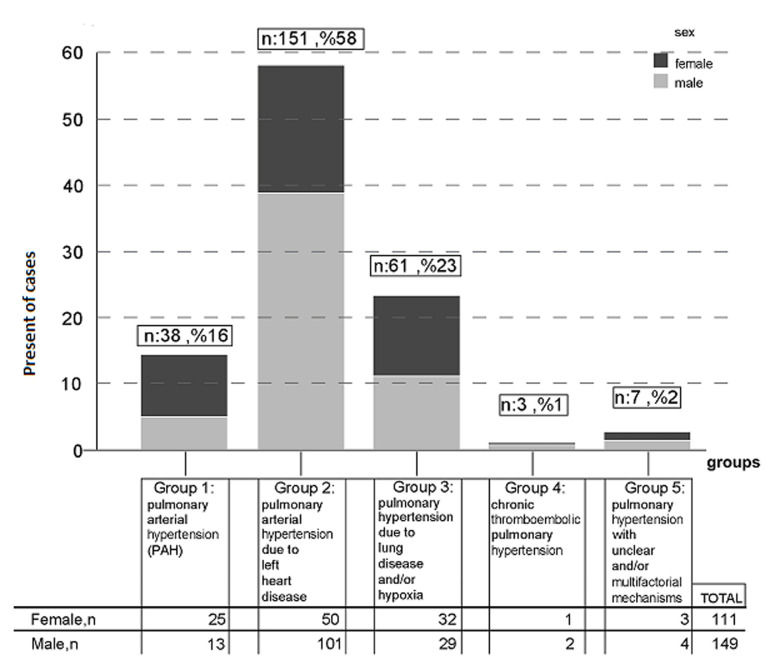
case distributions by subgroup classification of pulmonary hypertension

**PH distribution:** pulmonary hypertension (PH) due to left heart disease was the most common form (n=151, 58%), followed by PH due to lung disease (n=61, 23%), group 1 PH (n=38, 16%), group 5 PH (n=7, 2%), and CTEPH (n=3, 1%). Table 3 shows subgroup distributions of PH. In the subgroup analysis, congenital heart disease (n=34, 13.1%) was the most common cause of PAH. Heart failure with reduced EF (n=116, 44.6%) was the most frequent subgroup of PH due to left heart disease. In PH due to lung diseases, destructive pulmonary tuberculosis was the most common cause of PH (n=46, 17.7%). Group 2 PH was more common in males, while group 1 PH was more common in females. Figure 2 presents a detailed gender-based PH distribution.

**Table 3:** number of patients by subgroups of pulmonary hypertension

Subgroup	All patients (N=260)	%
**Group 1**	38	
Congenital heart disease	34	13.1%
Human immunodeficiency virus-associated pulmonary arterial hypertension	3	1.2%
Idiopathic pulmonary arterial hypertension	1	0.3%
**Group 2**	151	
Heart failure with reduced ejection fraction	116	44.6%
Rheumatic mitral valve disease	30	11.5%
Other valvular heart diseases	5	1.9%
**Group 3**	61	
Chronic obstructive pulmonary disease	15	5.8%
Post-Tuberculosis bronchiectasis	46	17.7%
Group 4	3	
**Group 5**	7	
Sicklecell anemia	2	0.8%
Others	5	1.9%

## Discussion

The aim of this study was to determine the prevalence and the etiological and demographic distribution of PH in the Somali population. The prevalence of PH was found to be 18%, which is much higher than that of the average of European countries [10,11]. In the Rotterdam study of 33181 patients, the prevalence of PH was found to be 2.6% [11]. In contrast, one study in Australia reported 9.1% [12]. Many diseases that may lead to PH are prevalent in Africa, particularly including tuberculosis, rheumatic heart disease, HIV infection, chronic hepatitis, hereditary hemoglobinopathies, and schistosomiasis [13-17]. In addition, failure to treat secondary causes of PH may also contribute to the high prevalence of PH in Africa. On the other hand, the prevalence of heart diseases has also been shown to be high in African countries [18,19], which may explain our finding that group 2 PH is the most common disease in participants. In a meta-analysis on pulmonary hypertension in Africa, left heart disease was found to be the most common etiologic cause, consistent with our study [20]. Untreated hypertensive heart disease is a leading cause of heart disease, which is highly common in the African population [21]. In our study, hypertension was detected in 21% of the patients. In addition, rheumatic valvular disease, which develops due to untreated streptococcal infections, is much more frequently seen in the African continent compared to developed countries [22]. In our study, the most common age group was 60-80 years and the mean age was 57 years, while in the Rotterdam study, the most common age group was 80 years and over and the mean age was 76.4 years [11].

This difference could be associated with a lower average life expectancy in Somalia and congenital heart diseases and other endemic causes of PH that affect the age distribution of the patients. In the Papuco study [23], which was a multinational study on PH epidemiology in Africa, group 2 PH constituted the largest group (69%), followed by group 1 PH (16%), group 3 PH (11%), group 4 PH (2%), and group 5 PH (2%). Although these findings were similar to our findings, the prevalence of group 3 PH was relatively higher in our study (23% vs.11%). This finding may be associated with a high rate of post-tuberculosis sequelae in a subgroup analysis (17%) in Somalia [24]. In addition, although the number of female patients was relatively higher in the Papuco study, the ratio of male patients (57.2%) was found to be higher in our study. In a large meta-analysis, the global prevalence of PH among HIV-infected adults was found 8.3% [25]. Pulmonary hypertension due to HIV is known to be common in the African population [26]. However, this entity was detected only in three (1.2%) patients in our study, which could be due to the religious sensitivities of the Somali community. Due to the same reason, no cases of PH secondary to schistosomiasis were detected in our study, although it is highly common in African countries. On the other hand, sickle cell anemia was detected in two patients, which was consistent with the literature on African countries [16]. In our study, PH due to congenital heart diseases was detected in 13.1% of the patients. In contrast, this rate was 5.7% in the study conducted by Nugunga *et al*. in Kenya [7] and was reported as 4.2% in the study conducted by Duffels *et al*. in Europe [27]. This difference could be attributed to higher fertility rates and insufficient pregnancy follow-up in African countries compared to European countries. On the other hand, heart failure with reduced EF, which was not related to the main subject of our study, was found in 116 patients, while an implantable cardioverter-defibrillator (ICD) was detected in only 3 (3.4%) patients.

In contrast, a study conducted in developed countries detected ICD in 10% of the patients [28]. Another important finding of our study was the high prevalence of significant pericardial effusion (38.4%). Meaningfully, significant pericardial effusion has been shown to be an independent predictor of mortality in PH [29,30]. In European studies, the mortality rate in patients with PH and pericardial effusion ranges between 25-30% [31,32]. Accordingly, the higher prevalence of significant pericardial effusion in our study could be ascribed to the fact that patients could not access specific treatments for PH and that the underlying diseases could not be adequately treated due to inadequate healthcare conditions in Somalia. Our study had several significant limitations. First and foremost, it was a single-center study and had a small number of patients. Second, it was highly difficult to reach reliable and complete clinical data of the patients since the number of patients with incomplete data was remarkably high and it took a long time to screen suitable patients; therefore, the number of patients who were eligible for inclusion was limited. Third, since there was no healthcare center in Somalia that could perform right heart catheterization, the diagnosis of PH could not be made with right heart catheterization in potential patients, which is the gold standard diagnostic tool in PH, and thus the diagnosis was made only through echocardiograms and the patients´ clinical records, which is likely to have caused problems such as missed diagnosis and overdiagnosis. Finally, the analysis of mortality associated with PH could not be conducted since there was no death registration and notification system in Somalia.

## Conclusion

The study indicated that the prevalence of PH in Somalia-Turkey Training and Research Hospital was relatively higher than the world average. The distribution characteristics of the disease can be related to the region-specific differences of the causative diseases. To reduce this high disease burden, the healthcare policymakers and planners in Somalia need to take necessary steps to improve the prevention, detection, and management of PH.

### What is known about this topic

- Pulmonary hypertension is an important cause of morbidity and mortality affecting millions of people worldwide;
- The distribution and rate of PH may differ between countries.

### What this study adds

- This is the first comprehensive study of PH from Somalia that reports the frequency and distribution of PH among the Somalia population;
- The pattern and prevalence of PH is different among the Somalia population as compared to other countries.

## References

[1] Maron BA, Brittain EL, Choudhary G, Gladwin MT (2018). Redefining pulmonary hypertension. Lancet Respir Med.

[2] Simonneau G, Montani D, Celermajer DS, Denton CP, Gatzoulis MA, Krowka M (2019). Haemodynamic definitions and updated clinical classification of pulmonary hypertension. Eur Respir J.

[3] Hurdman J, Condliffe R, Elliot CA, Davies C, Hill C, Wild JM (2012). ASPIRE registry: assessing the spectrum of pulmonary hypertension identified at a referral centre. Eur Respir J.

[4] Stewart S, Mocumbi AO, Carrington MJ, Pretorius S, Burton R, Sliwa K (2011). A not-so-rare form of heart failure in urban black Africans: pathways to right heart failure in the Heart of Soweto Study cohort. Eur J Heart Fail.

[5] Dzudie A, Dzekem BS, Ojji DB, Kengne AP, Mocumbi AO, Sliwa K (2020). Pulmonary hypertension in low-and middle-income countries with focus on sub-Saharan Africa. Cardiovasc Diagn Ther.

[6] Dzudie A, Dzekem BS, Tchoumi CT, Aminde LN, Mocumbi AO, Abanda M (2018). Pulmonary hypertension as seen in a rural area in sub-Saharan Africa: high prevalence, late clinical presentation and a high short-term mortality rate during follow up. Cardiovasc J Afr.

[7] Ngunga M, Mansur Abeid A, Mohamed J, Barasa A (2020). Long-term outcomes and factors associated with mortality in patients with moderate to severe pulmonary hypertension in Kenya. Glob Heart.

[8] Warsame A, Handuleh J, Patel P (2016). Prioritization in Somali health system strengthening: a qualitative study. Int Health.

[9] Zoghbi WA, Adams D, Bonow RO, Enriquez-Sarano M, Foster E, Grayburn PA (2017). Recommendations for noninvasive evaluation of native valvular regurgitation: a report from the American society of echocardiography developed in collaboration with the society for cardiovascular magnetic resonance. J Am Soc Echocardiogr.

[10] Hoeper MM, Humbert M, Souza R, Idrees M, Kawut SM, Sliwa-Hahnle K (2016). A global view of pulmonary hypertension. Lancet Respir Med.

[11] Moreira EM, Gall H, Leening MJ, Lahousse L, Loth DW, Krijthe BP (2015). Prevalence of pulmonary hypertension in the general population: the Rotterdam study. PLoS One.

[12] Strange G, Playford D, Stewart S, Deague JA, Nelson H, Kent A (2012). Pulmonary hypertension: prevalence and mortality in the Armadale echocardiography cohort. Heart.

[13] Cox H, Hughes J, Daniels J, Azevedo V, McDermid C, Poolman M (2014). Community-based treatment of drug-resistant tuberculosis in Khayelitsha, South Africa. Int J Tuberc Lung Dis.

[14] Bigna JJ, Nansseu JR, Um LN, Noumegni SR, Sime PS, Aminde LN (2016). Prevalence and incidence of pulmonary hypertension among HIV-infected people in Africa: a systematic review and meta-analysis. BMJ Open.

[15] Mohd Hanafiah K, Groeger J, Flaxman AD, Wiersma ST (2013). Global epidemiology of hepatitis C virus infection: new estimates of age-specific antibody to HCV seroprevalence. Hepatology.

[16] Aliyu ZY, Kato GJ, Taylor J 6th, Babadoko A, Mamman AI, Gordeuk VR (2008). Sickle cell disease and pulmonary hypertension in Africa: a global perspective and review of epidemiology, pathophysiology, and management. Am J Hematol.

[17] Graham BB, Bandeira AP, Morrell NW, Butrous G, Tuder RM (2010). Schistosomiasis-associated pulmonary hypertension: pulmonary vascular disease: the global perspective. Chest.

[18] Sliwa K, Wilkinson D, Hansen C, Ntyintyane L, Tibazarwa K, Becker A (2008). Spectrum of heart disease and risk factors in a black urban population in South Africa (the heart of Soweto study): a cohort study. Lancet.

[19] Stewart S, Wilkinson D, Hansen C, Vaghela V, Mvungi R, McMurray J (2008). Predominance of heart failure in the heart of Soweto study cohort: emerging challenges for urban African communities. Circulation.

[20] Bigna JJ, Noubiap JJ, Nansseu JR, Aminde LN (2017). Prevalence and etiologies of pulmonary hypertension in Africa: a systematic review and meta-analysis. BMC Pulm Med.

[21] Dzudie A, Kengne AP, Muna WF, Ba H, Menanga A, Kouam Kouam C (2012). Prevalence, awareness, treatment and control of hypertension in a self-selected sub-Saharan African urban population: a cross-sectional study. BMJ Open.

[22] Engel ME, Haileamlak A, Zühlke L, Lemmer CE, Nkepu S, van de Wall M (2015). Prevalence of rheumatic heart disease in 4720 asymptomatic scholars from South Africa and Ethiopia. Heart.

[23] Thienemann F, Dzudie A, Mocumbi AO, Blauwet L, Sani MU, Karaye KM (2016). The causes, treatment, and outcome of pulmonary hypertension in Africa: insights from the Pan African pulmonary hypertension cohort (PAPUCO) registry. Int J Cardiol.

[24] Lillebaek T, Andersen AB, Bauer J, Dirksen A, Glismann S, de Haas P (2001). Risk of *Mycobacterium tuberculosis*transmission in a low-incidence country due to immigration from high-incidence areas. J Clin Microbiol.

[25] Bigna JJ, Nansseu JR, Noubiap JJ (2019). Pulmonary hypertension in the global population of adolescents and adults living with HIV: a systematic review and meta-analysis. Sci Rep.

[26] Aminde LN, Dzudie A, Kengne AP, Ndjebet J, Mapoh S, Kuelang X (2017). Gender disparities in pulmonary hypertension at a tertiary centre in Cameroon. S Afr Med J.

[27] Duffels MG, Engelfriet PM, Berger RM, van Loon RL, Hoendermis E, Vriend JW (2007). Pulmonary arterial hypertension in congenital heart disease: an epidemiologic perspective from a Dutch registry. Int J Cardiol.

[28] Schrage B, Uijl A, Benson L, Westermann D, StÃ¥hlberg M, Stolfo D (2019). Association between use of primary-prevention implantable cardioverter-defibrillators and mortality in patients with heart failure: a prospective propensity score-matched analysis from the Swedish heart failure registry. Circulation.

[29] Honeycutt GR, Safdar Z (2013). Pulmonary hypertension complicated by pericardial effusion: a single center experience. Ther Adv Respir Dis.

[30] Benza RL, Miller DP, Gomberg-Maitland M, Frantz RP, Foreman AJ, Coffey CS (2010). Predicting survival in pulmonary arterial hypertension: insights from the registry to evaluate early and long-term pulmonary arterial hypertension disease management (REVEAL). Circulation.

[31] Fenstad ER, Le RJ, Sinak LJ, Maradit-Kremers H, Ammash NM, Ayalew AM (2013). Pericardial effusions in pulmonary arterial hypertension: characteristics, prognosis, and role of drainage. Chest.

[32] Shimony A, Fox BD, Langleben D, Rudski LG (2013). Incidence and significance of pericardial effusion in patients with pulmonary arterial hypertension. Can J Cardiol.

